# Supplementary material for: Analysis of Chemical Composition and Antioxidant Activity of Idesia polycarpa Pulp Oil from Five Regions in China
Source: Foods. 2023 Mar 15;12(6):1251. doi: 10.3390/foods12061251 (PMC10048772; doi:10.3390/foods12061251)
Supplement: Supplementary file 1 [file foods-12-01251-s001.zip › foods-2204733-Supplementary.pdf]

Table S1 Fitting curves of bioactive compounds.

Components	Regression equation	R^2	Linearity range (μg/mL)
α-tocopherol	Y = 9366.5 X + 7205.1	0.9986	2.5860-103.4397
β-tocopherol	Y = 9257.6 X + 757.09	0.9984	0.4016-16.0648
γ-tocopherol	Y = 9292.8 X + 48558	0.9984	15.8762-635.0409
δ-tocopherol	Y = 9399 X + 13782	0.9985	6.1362-245.4461
Polyphenol	Y = 0.0958 X + 0.0293	0.9990	1-15

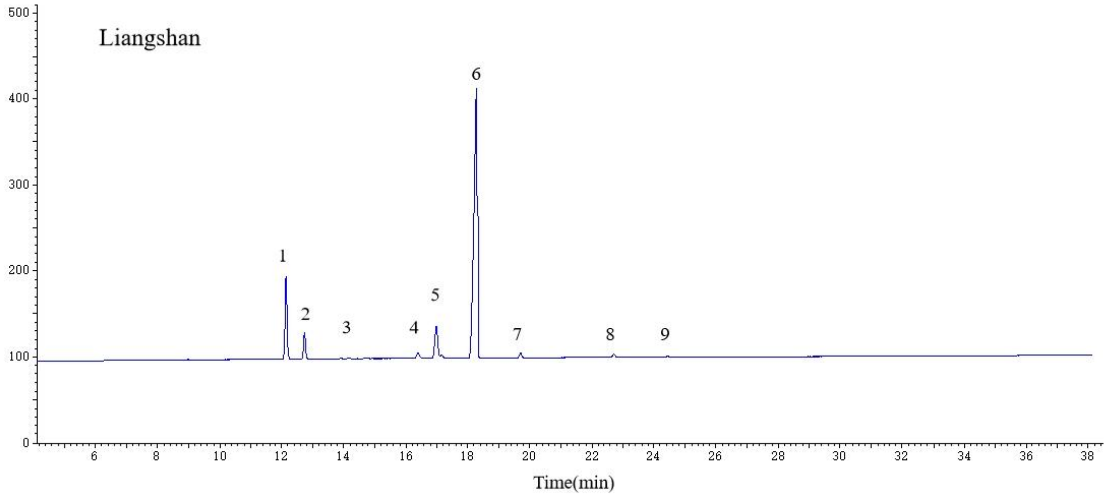
Liangshan
500
400
300
200
100
0
1
2
3
4
5
6
7
8
9
6 8 10 12 14 16 18 20 22 24 26 28 30 32 34 36 38
Time(min)

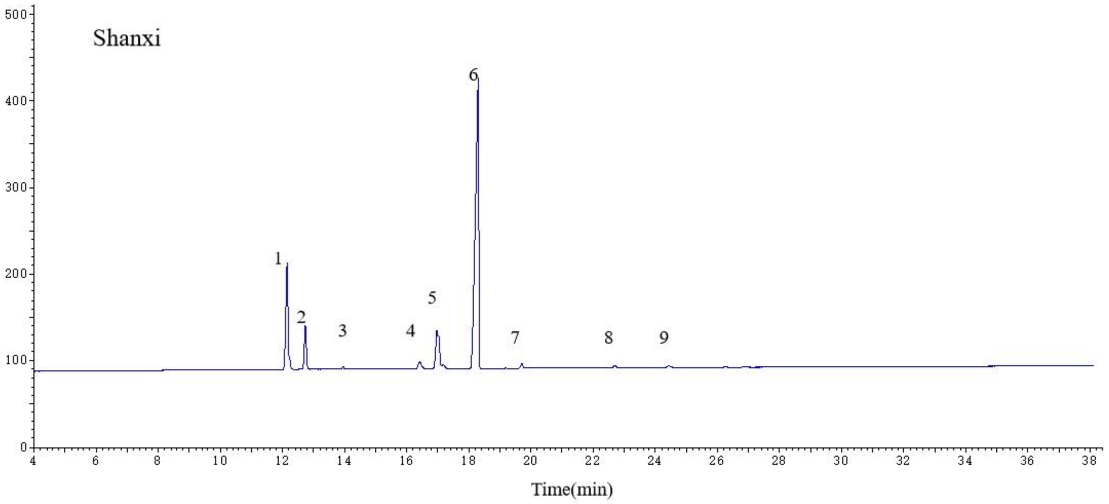
Shanxi
500
400
300
200
100
0
1
2
3
4
5
6
7
8
9
4 6 8 10 12 14 16 18 20 22 24 26 28 30 32 34 36 38
Time(min)

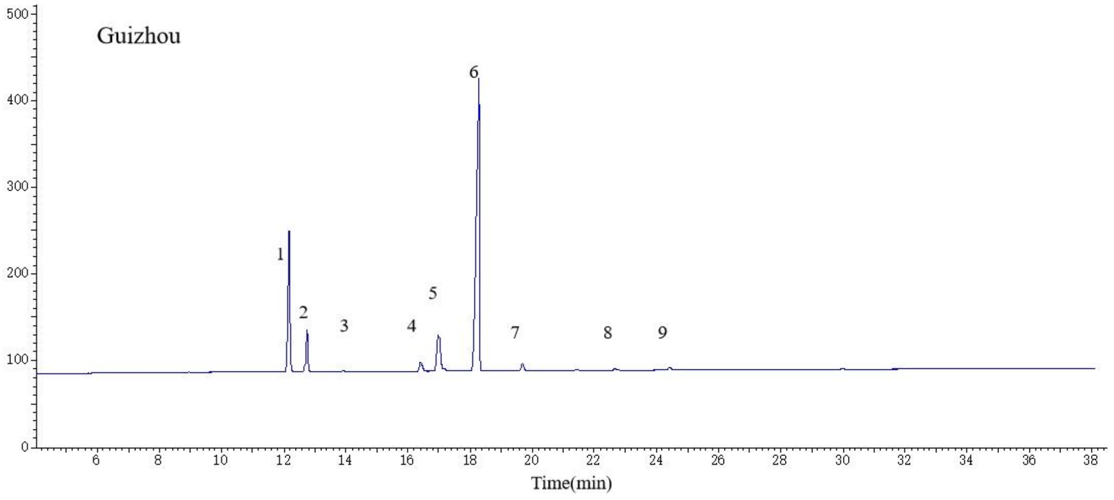
Guizhou
500
400
300
200
100
0
1
2
3
4
5
6
7
8
9
6 8 10 12 14 16 18 20 22 24 26 28 30 32 34 36 38
Time(min)

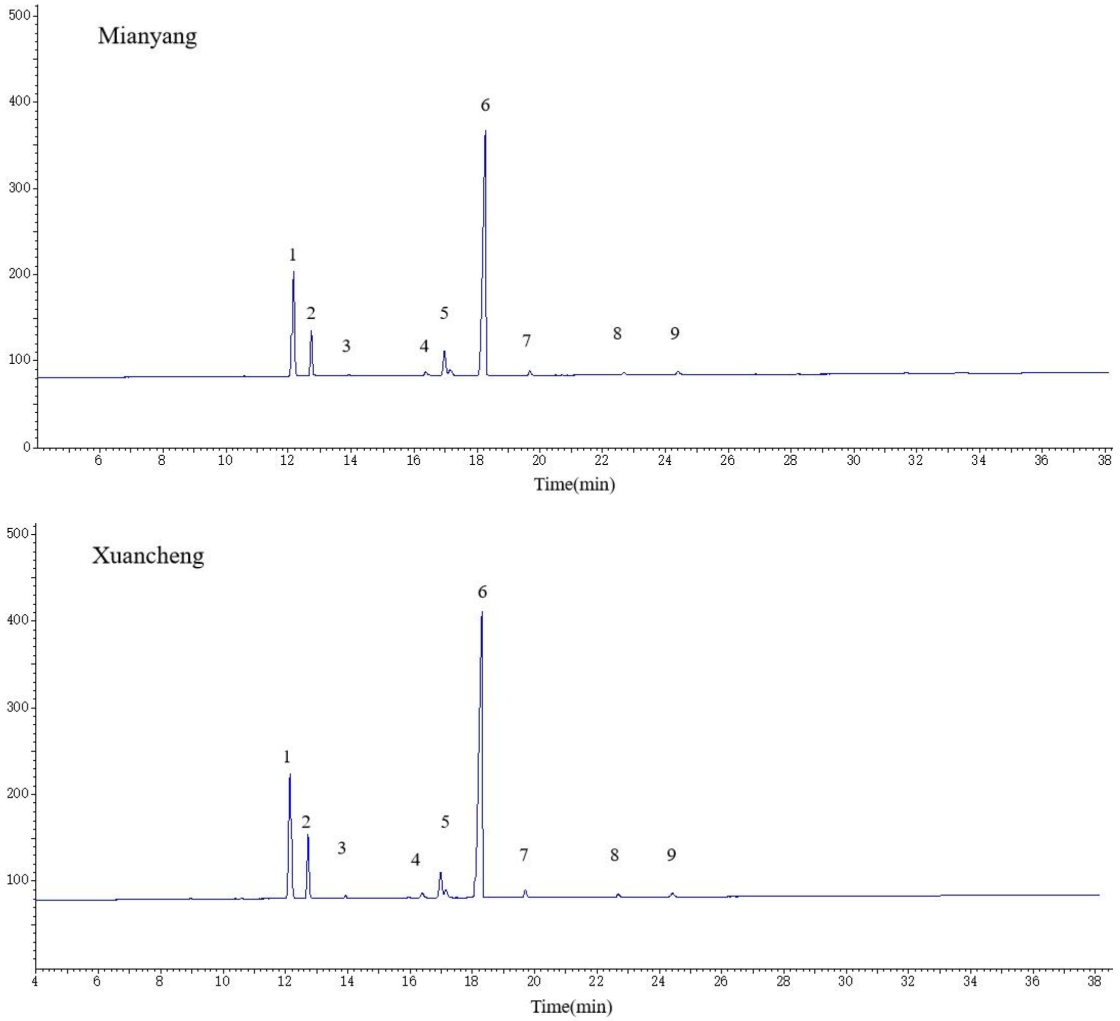

Figure S1. Fatty acid composition gas chromatogram of *Idesia polycarpa* pulp oil from five regions. 1- palmitic acid, 2-palmitoleic acid, 3-heptadecanoic acid, 4- stearic acid, 5-oleic acid, 6-linolei cacid, 7-linolenic acid, 8-arachidic acid, 9- heneicosanoic acid.

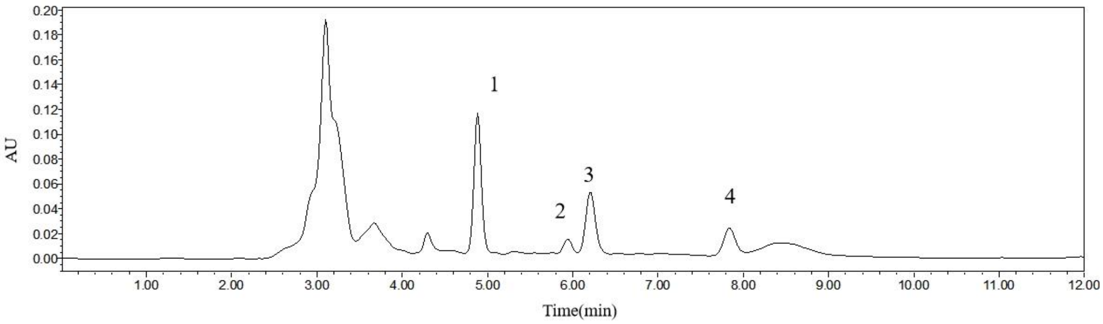

Figure S2. HPLC of tocopherols in *Idesia polycarpa* pulp oil. 1-α-tocopherol, 2-β-tocopherol, 3-γ-tocopherol, 4-δ-tocopherol.

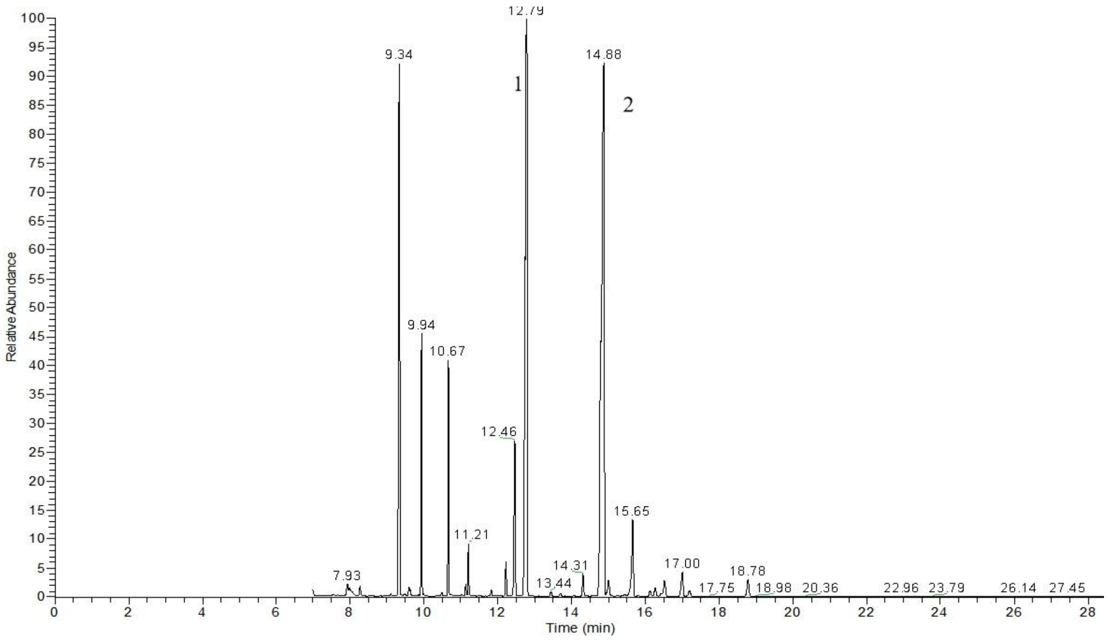

Figure S3. Total ion chromatogram of β-sitosterol in *Idesia polycarpa* pulp oil.
1-5α-cholestane, 2-β-sitosterol.
